# Three-dimensional-printed polycaprolactone scaffolds with interconnected hollow-pipe structures for enhanced bone regeneration

**DOI:** 10.1093/rb/rbac033

**Published:** 2022-05-30

**Authors:** Jiahua Duan, Dong Lei, Chen Ling, Yufeng Wang, Zhicheng Cao, Ming Zhang, Huikang Zhang, Zhengwei You, Qingqiang Yao

**Affiliations:** Department of Orthopaedic Surgery, Nanjing First Hospital, Nanjing Medical University, Nanjing 210006, China; State Key Laboratory for Modification of Chemical Fibers and Polymer Materials, College of Materials Science and Engineering, Institute of Functional Materials, Donghua University, Shanghai 201620, China; Research Base of Textile Materials for Flexible Electronics and Biomedical Applications (China Textile Engineering Society), Shanghai Engineering Research Center of Nano-Biomaterials and Regenerative Medicine, Shanghai 201620, China; Department of Cardiology, Shanghai 9th People’s Hospital, Shanghai Key Laboratory of Tissue Engineering, School of Medicine, Shanghai Jiao Tong University, Shanghai 200011, China; Department of Orthopaedic Surgery, Nanjing First Hospital, Nanjing Medical University, Nanjing 210006, China; Department of Orthopaedic Surgery, Nanjing First Hospital, Nanjing Medical University, Nanjing 210006, China; Department of Orthopaedic Surgery, Nanjing First Hospital, Nanjing Medical University, Nanjing 210006, China; Department of Orthopaedic Surgery, Nanjing First Hospital, Nanjing Medical University, Nanjing 210006, China; Department of Orthopaedic Surgery, Nanjing First Hospital, Nanjing Medical University, Nanjing 210006, China; State Key Laboratory for Modification of Chemical Fibers and Polymer Materials, College of Materials Science and Engineering, Institute of Functional Materials, Donghua University, Shanghai 201620, China; Research Base of Textile Materials for Flexible Electronics and Biomedical Applications (China Textile Engineering Society), Shanghai Engineering Research Center of Nano-Biomaterials and Regenerative Medicine, Shanghai 201620, China; Department of Orthopaedic Surgery, Nanjing First Hospital, Nanjing Medical University, Nanjing 210006, China

**Keywords:** three-dimensional printing, interconnected hollow-pipe structure, bone regeneration, vascularization, polycaprolactone

## Abstract

Three-dimensional (3D)-printed scaffolds are widely used in tissue engineering to help regenerate critical-sized bone defects. However, conventional scaffolds possess relatively simple porous structures that limit the delivery of oxygen and nutrients to cells, leading to insufficient bone regeneration. Accordingly, in the present study, perfusable and permeable polycaprolactone scaffolds with highly interconnected hollow-pipe structures that mimic natural micro-vascular networks are prepared by an indirect one-pot 3D-printing method. *In vitro* experiments demonstrate that hollow-pipe-structured (HPS) scaffolds promote cell attachment, proliferation, osteogenesis and angiogenesis compared to the normal non-hollow-pipe-structured scaffolds. Furthermore, *in vivo* studies reveal that HPS scaffolds enhance bone regeneration and vascularization in rabbit bone defects, as observed at 8 and 12 weeks, respectively. Thus, the fabricated HPS scaffolds are promising candidates for the repair of critical-sized bone defects.

## Introduction

Bone is a highly branched vascularized system that can deliver oxygen and nutrients to cells and remove metabolites in the repair of damaged tissues [1, 2]. Rapid vascularization not only allows the delivery of oxygen and nutrients for cell proliferation but also supplies required ions and growth factors for bone regeneration [3, 4]. Therefore, vascularization plays a pivotal role in bone regeneration. Clinically, the reconstruction of critical-sized bone defects is a worldwide problem [5]. Current therapies, which include allografting, autografting and other bone transport techniques, are limited in availability and may lead to the development of chronic pain [6, 7]. Accordingly, three-dimensional (3D) bioprinting technology has recently emerged as a potential strategy for the repair of critical-sized bone defects resulting from infections, surgery, trauma and congenital malformations [8]. However, conventional scaffolds possess relatively simple porous structures and thus exhibit poor delivery of nutrients and oxygen, low porosity and poor vascularization performance, thus limiting the generation of new bone inside the scaffold [9–12]. Moreover, due to the limited diffusion of oxygen and nutrients, the inner regions of larger 3D-printed constructs are prone to necrosis, which induces an inflammatory response *in vivo* [13, 14].

To facilitate bone regeneration, studies focusing on the fabrication of vascular-like constructs using various microfabrication techniques, including sacrificial molding, laser-piercing and wire-array templating, have been conducted [15–17]. Accordingly, these different groups have demonstrated that scaffolds with hollow channels can promote vascularization and bone regeneration in critical-sized bone defects [16–18]. However, most of these scaffolds achieved limited success due to poor fabrication efficiency, low porosity, and problems associated with biodegradation, and biocompatibility [19–21]. In addition, these approaches cannot be easily used to make 3D constructs and often require multiple complex steps. Several studies focused on the creation of hollow channels in scaffolds using modified coaxial 3D-printing methods to improve bone regeneration [15, 22–25]. However, the hollow channels in these scaffolds were partially interconnected in different layers. Furthermore, these channel surfaces was often smooth or the porous structure in the channel surface was not controllable. Therefore, they did not allow perfusion throughout the whole scaffold, which further limited their success in bone regeneration. Moreover, previous studies showed that microfluidic systems containing microchannels could promote the formation of rudimentary vasculatures *in vitro* [23, 26, 27]. However, such systems are constructed from hydrogels, which are difficult to anastomose to the host, degrade rapidly after implantation and have poor mechanical properties [28, 29].

In this study, we attempted to combine the use of one-pot 3D-printed sacrificial caramel templates and polymer coating with integrated phase separation to prepare a highly interconnected hollow-pipe-structured (HPS) scaffold for regenerating bone defects. These HPS scaffolds mimic the perfusable and permeable channel structure of natural micro-vascular networks to provide enhanced delivery of oxygen and nutrients to the whole scaffold. More importantly, we used the ubiquitous phase separation mechanism to readily introduce extensive and controllable micropores in the channel walls, which is seldom reported previously. The as-prepared scaffolds possessed distinctly improved porosity for cell adhesion and proliferation. In particular, our *in vitro* studies demonstrated that HPS scaffolds were beneficial for osteogenesis and angiogenesis, while *in vivo* studies revealed that HPS scaffolds enhanced bone tissue formation and vascularization of rabbit bone defects. Moreover, more bone tissues were observed in the inner regions of HPS scaffolds than in corresponding regions of non-hollow-pipe-structured (NHPS) scaffolds. Thus, we have developed a smart scaffold that can effectively regenerate critical-sized bone defects and may be applied clinically in the future.

**Figure:**
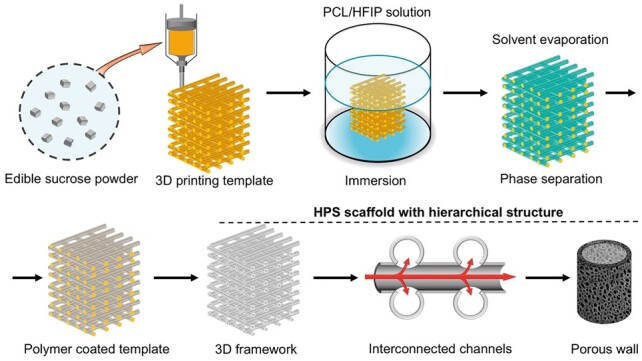


## Materials and methods

### Fabrication and characterization of HPS and NHPS scaffolds

**Figure 1.**
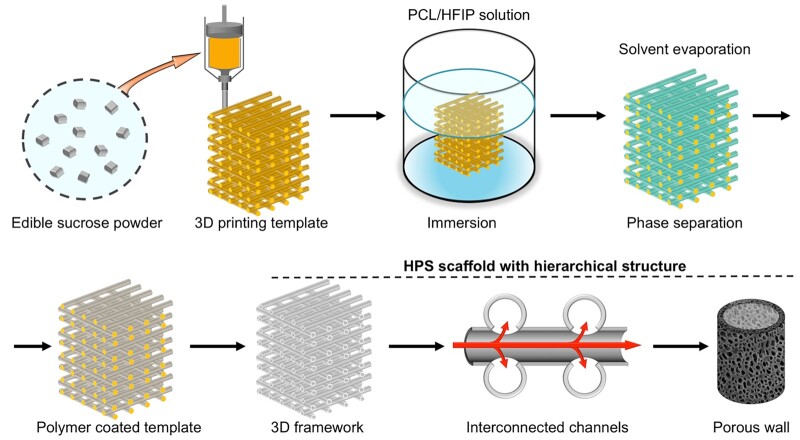
Schematic of the design and fabrication of HPS scaffold with hierarchical architectures.

HPS scaffolds were generated by the indirect one-pot 3D-printing method in a similar strategy developed in our previous study [30, 31] (Fig. 1). First, a caramel-based template was printed using fused deposition modeling (FDM). Sucrose was preheated to 150°C for 30 min in a 3D printer (HTS-400; Fochif Mechatronics Technology, China), and the caramel ink was printed into 12-layer constructs at 130–135°C. The center-to-center distance between the filaments was 1.3 mm with 0°/90° lay-down patterning between two successive layers; the height of every layer was 0.5 mm; and the nozzle size was 20 G. Polycaprolactone (PCL; *Mn* 80 000 g mol^−1^, Sigma-Aldrich) was dissolved in hexafluoroisopropanol (HFIP; analytical grade, >99%, Sigma-Aldrich) at 4% (*w/v*). The caramel-based template was soaked in the PCL solution for 1 min, and the HFIP solvent was evaporated for 30 min under ambient conditions. Then, the PCL-coated template was soaked in distilled water for 6 h to remove the caramel and residual solvent. The water was replaced every 1 h. Next, the PCL HPS scaffold was lyophilized. NHPS scaffolds were directly printed by FDM at 110°C with PCL using the same parameters.

To observe the detailed structures of HPS and NHPS scaffolds, they were subjected to scanning electron microscopy (SEM; JSM-5600LV, Jeol Ltd, Japan) in the side- and top-view orientations. First, scaffolds were sputter-coated with gold. Cutting the scaffold transversely revealed the filament cross-section. The porosities of the scaffolds were determined using a previously reported liquid displacement method [32].

### *In vitro* analysis

#### CCK-8 assay

Rabbit bone marrow stem cells (BMSCs) were isolated via a previously described method [33]. The proliferation of BMSCs was evaluated by Cell Counting Kit-8 (CCK-8) assay. Briefly, 6 × 10^5^ BMSCs were seeded vertically on the surfaces of scaffolds in 24-well plates and then cultured in Dulbecco’s modified Eagle medium (DMEM) (Thermo Fisher Scientific, USA) containing 10% (v/v) fetal calf serum (Thermo Fisher Scientific, USA) and 1% (v/v) penicillin–streptomycin (Thermo Fisher Scientific, USA). CCK-8 assays were conducted 1, 3, 5 and 7 days after incubation. When tested, cell-seeded scaffolds were incubated in 10% CCK-8 solution (CCK-8; Biomake, China) at 37°C for 30 min. The solution absorbance at 450 nm was then measured with a microplate reader (Infinite F50, TECAN, Switzerland).

#### Live/dead staining

The viabilities of the cells on the scaffolds were determined using live/dead kits (Invitrogen, UK). Briefly, 6 × 10^5^ BMSCs were seeded vertically on the surfaces of scaffolds in 24-well plates and then cultured in DMEM containing 10% (v/v) fetal calf serum and 1% (v/v) penicillin–streptomycin for 3 or 7 days. Then, 2 × 10^−6 ^mol calcein AM and 4 × 10^−6 ^mol EthD-1 solutions were added to 2 ml phosphate buffered saline (PBS), and 1000 μL of this solution was added to the cell-seeded scaffolds in 24-well plates. After incubating for 30 min at room temperature, the scaffolds were gently washed with PBS solution. Images were obtained by laser confocal microscopy (LSM700, Zeiss, Germany). The viable cells appeared green, whereas dead cells appeared red.

#### DAPI staining

BMSCs (6 × 10^5^) were seeded vertically on the surfaces of scaffolds in 24-well plates and then cultured in DMEM containing 10% (v/v) fetal calf serum and 1% (v/v) penicillin–streptomycin. After culturing for 3 days, the cell nuclei were stained with DAPI (Beyotime, China) and incubated for 1 h at room temperature. Fluorescence images were captured using a fluorescence microscope (ZEISS, Axio, Germany). Cell numbers were determined using Image J software (National Institutes of Health, USA).

#### SEM analysis of scaffolds

BMSCs cultured in scaffolds were observed by SEM. First, 6 × 10^5^ BMSCs were seeded vertically on scaffolds in 24-well plates and then cultivated in DMEM containing 10% (v/v) fetal calf serum and 1% (v/v) penicillin–streptomycin for 7 days. Next, the cell-seeded scaffolds were immersed in 4% paraformaldehyde for 1 h. Before SEM observation (S4800; HITACHI, Japan), the scaffolds were sputter-coated with gold.

#### Alkaline phosphatase activity

The differentiation of BMSCs in the early stage of osteogenesis was evaluated by alkaline phosphatase (ALP) activity. First, 6 × 10^5^ BMSCs were seeded vertically on scaffolds in 24-well plates and then cultivated in an osteoinductive medium containing 50 µg/ml ascorbic acid, 10 mM β-glycerol phosphate and 10^−8^M dexamethasone (Cyagen Biosciences, America) for 7 or 14 days. Then, BMSCs were lysed using 100 μL of RIPA lysis buffer. The ALP activity of the BMSCs was evaluated with an Alkaline Phosphatase Assay Kit (Beyotime, China) according to the manufacturer’s protocol. Briefly, BMSCs were incubated with the assay buffer and the samples were centrifuged to eliminate insoluble substances. Then, p-nitrophenylphosphatase liquor was mixed with the cell lysate, and the mixed solution was incubated at 37°C for 30 min. The concentration of p-nitrophenyl was determined by measuring the absorbance at 405 nm using a microplate reader (Synergy 2; BioTek, USA) and analyzing the result using a standard curve.

#### Quantitative real-time polymerase chain reaction analysis

To evaluate the mRNA expression levels of osteogenic genes (*COL-I, OCN, OPN* and* RUNX2*), BMSCs were processed for total RNA extraction using an RNA prep Micro Kit (TaKaRa, Japan) at 7 or 14 days. First, 6 × 10^5^ BMSCs were seeded vertically on the surfaces of scaffolds and then cultivated in an osteoinductive medium. Then, the mRNA expression levels of osteogenic genes were assessed by quantitative real-time polymerase chain reaction (RT-PCR) analysis. Specifically, total RNA was extracted from the BMSCs using the Trizol reagent (Ambion, CA) following the manufacturer’s instructions. Thereafter, complementary DNA was synthesized using All-In-One RT MasterMix (ABM, CA). After reverse transcription, RT-PCR was performed using EvaGreen 2 × qPCR MasterMix (ABM, CA) on a Light Cycler 480 II (Roche, CHE).

To evaluate the expression of angiogenesis genes (*HIF1-α* and *VEGF*), human umbilical vein endothelial cells (HUVECs) were purchased from AllCells. First, 6 × 10^5^ HUVECs were seeded vertically on the scaffolds and cultured in an endothelial basal medium. Then, the same procedure used to evaluate osteogenic genes was also used for angiogenesis-specific genes. All primer sequences were designed using the software Primer 5.0 from the NCBI database. Primer sequences: rabbit, GAPDH 5′-TCACCATCTTCCAGGAGCGA-3′ and 5′-CACAATGCCGAAGTGGTCGT-3′; RUNX2 5′-TCAGGCATGTCCCTCGGTAT-3′ and 5′-TGGCAGGTAGGTATGGTAGTGG-3′; OPN 5′-CACCATGAGAATCGCCGT-3′ and 5′-CGTGACTTTGGGTTTCTACGC-3′; OCN 5′-CCGGGAGCAGTGTGAGCTTA-3′ and 5′-AGGCGGTCTTCAAGCCATACT-3′; COLI 5′-CTTCTGGCCCTGCTGGAAAGGATG-3′ and 5′-CCCGGATACAGGTTTCGCCAGTAG-3′; HIF-1α 5′-CCATGTGACCATGAGGAAAT-3′ and 5′-CGGCTAGTTAGGGTACACTT-3′; VEGF 5′-CTACCTCCACCATGCCAAGT-3′ and 5′-AGCTGCGCTGATAGACATCC-3′.

#### Immunofluorescence staining in vitro

BMSCs and HUVECs were cultured in osteoinductive medium and endothelial basal medium, respectively, in the presence of HPS and NHPS scaffolds, as described above. After incubating for 7 days, cells were fixed with 4% paraformaldehyde solution and washed with PBS. Next, cells were permeabilized with 0.2% (*v/v*) Triton X-100 for 15 min and nonspecific binding was blocked with 10% goat serum solution (Invitrogen, US). Then, BMSCs with the primary antibodies OCN (ab13420, Abcam, 1:100), OPN (ab8448, Abcam, 1:100) and HUVECs with VEGF (ab115805, Abcam, 1:100) and CD31 (ab28364, Abcam, 1:100) were incubated for 1 h at room temperature. After primary antibody incubation, cells were washed with PBS and incubated with appropriate Alexa-Fluor-coupled secondary antibodies (Life Tech, USA, 1:400) for 1 h at room temperature. The images were captured using a fluorescent microscope (ZEISS, Axio, Germany).

### *In vivo* analysis

#### Bone defect model

All animal experiments were conducted following the relevant laws and guidelines. After receiving the approval of the ethics committee of Nanjing Medical University, New Zealand, rabbits were purchased from Nanjing First Hospital Animal Center for *in vivo* experiments. Both the posterior limbs of rabbits were used for defects. Defects were treated with HPS scaffolds, NHPS scaffolds or left untreated (no scaffold) as a control (CTR). First, rabbits were anesthetized through intramuscular injection. After exposing the femoral epicondyle by lateral incision, critical size defects (6 mm diameter, 6 mm height) were transversally created at the femoral epicondyle. HPS or NHPS scaffolds with a diameter of 6 mm and a height of 6 mm were implanted into the defects. The rabbits were sacrificed 8 or 12 weeks after implantation for *in vivo* evaluation.

#### Micro-CT analysis

The samples were removed from the rabbits for Micro-CT analysis (Siemens Inveon, Siemens Medical Solution, Germany) immediately after sacrifice. New bone regeneration was distinguished from other tissues by gray value with a threshold of 1500–2800. The new bone volume fractions in the defects (BV/TV, new bone area/total area) and the bone mineral densities (BMDs) of the samples were determined.

#### Histological analysis

All samples were immersed in 4% paraformaldehyde for 48 h and then decalcified in 5% formic acid for 3 days. Then, these samples were embedded in paraffin and cut into 5-μm-thick sections using a microtome. For histological analysis, hematoxylin and eosin staining was performed on the sections. For the immunofluorescence staining analysis of *in vivo* samples, bone sections were incubated overnight with primary antibodies of HIF-1α antibody (Beyotime, China), CD31antibody (Beyotime, China) and DAPI (Beyotime, China). The slides were analyzed using a fluorescent stereomicroscope and digital camera (Olympus BX-53, Tokyo, Japan). Signal intensities were determined using Image J software (National Institutes of Health, USA).

### Statistical analysis

All data were expressed as mean ± standard deviations and analyzed using one-way analysis of variance. *P *<* *0.05 was considered statistically significant.

## Results and discussion

### Fabrication and characterization of HPS scaffolds

**Figure 2.**
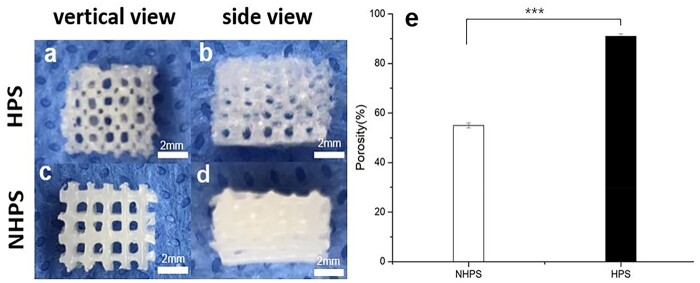
Morphology and porosity of the 3D-printed scaffolds. (**a** and **b**) Top- and side-view, respectively, of an HPS scaffold. (**c** and **d**) Top- and side-view, respectively, of an NHPS scaffold. (**e**) Porosities of the scaffolds (*n* = 6 for each group, ***P < 0.001).

**Figure 3.**
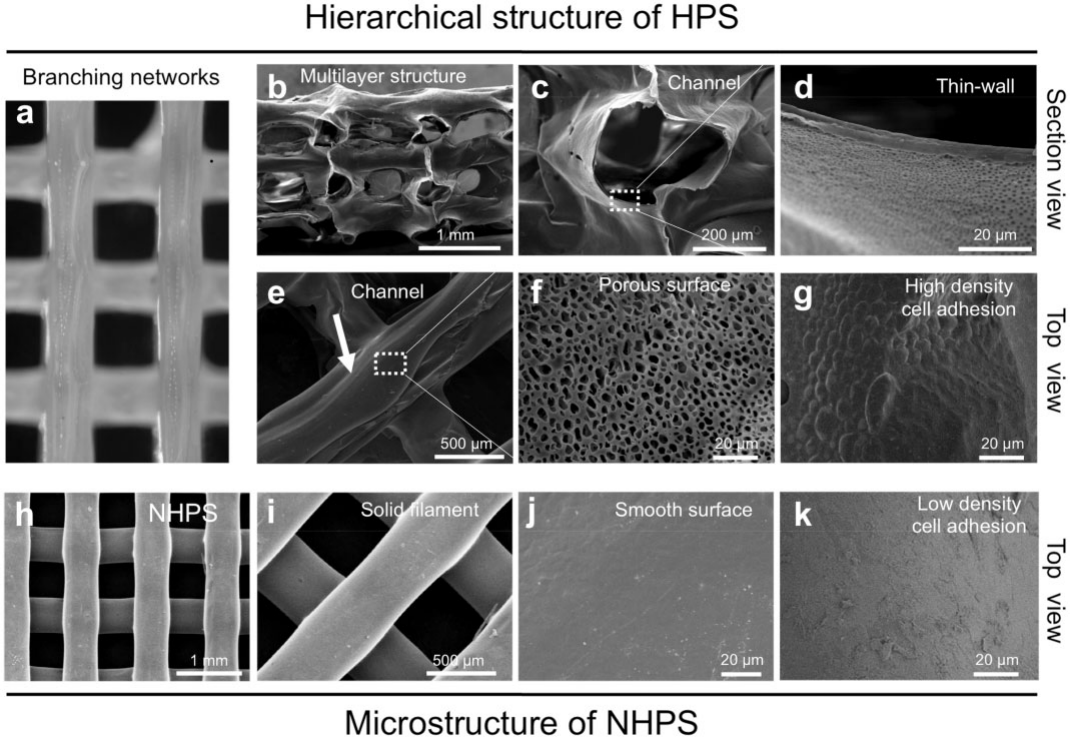
Microstructure of 3D-printing scaffolds. (**a**–**g**) Three-level hierarchical microstructures of HPS in top and sectional views. (a, optical image; b–g, SEM). (a and b) Primary structure: branching network was built by vertically stacking hollow channels; (c and e) secondary structure: interconnected and hollow channels with smooth junctions. (d and f) Tertiary structure: porous and thin channel walls enabled mass exchange inside and outside of the channels. (g) A large quantity and high density of BMSCs attached inside of hollow channel of HPS scaffolds. (**h**–**k**) SEMs of NHPS in top view. (h) Multilayer structure; (i) solid and cylindrical filament; (j) smooth surface of filament and (k) few BMSCs attached on the surface of NHPS scaffolds.

HPS scaffolds have controllable multi-stage bionic structures, including an interconnected branch network and permeable channel walls with microporous structures (Fig. 2). The SEM images of HPS scaffolds showed that the frameworks had three-level hierarchical structures (Fig. 3). The primary structure of the framework was formed by vertically stacked hollow channels (Fig. 3a and b). The crisscross pattern of the HPS scaffold was closely aligned with that of the caramel template. The secondary microchannel structure was continuous and connected seamlessly to adjacent perpendicular layers at numerous cross-points (Fig. 3e). Removal of the caramel template gave rise to integrated and interconnected microchannels that support physiologically relevant perfusion to mimic microvasculature for mass transfer in three dimensions (Fig. 3c). Furthermore, the micropore-rich tertiary structure, which was generated by phase separation during solvent evaporation, was distributed throughout the thin channel walls (Fig. 3d and f). These micropores showed relatively uniform pore sizes of 5.48 ± 1.80 μm, and the thickness of hollow channel wall was 13.76 ± 3.18 μm. The phase separation process has been widely used to build porous structures. In a previous study, we investigated the coating process via the phase separation mechanism in detail including different molecular weight, concentrations and solvents of PCL. These factors had significant effects on the morphology of the resultant scaffolds [30]. The permeable porous channel walls enable the osmotic exchange of nutrients and waste between the inside and pores of the channels.

A liquid displacement method was used to measure the porosities of HPS and NHPS scaffolds, revealing porosities of 91% and 55%, respectively (Fig. 2e). Thus, the porosity of HPS scaffolds was significantly enhanced compared to that of NHPS scaffolds. HPS and NHPS scaffolds had similar structures in terms of frame structure and stacking layers (Fig. 3h), therefore, the porosities resulting from the interstitial space between the channels of HPS scaffold and between the filaments of NHPS scaffold were similar. However, unlike the solid filaments and smooth surface of NHPS scaffold (Fig. 3i and j), the channels of HPS scaffold were hollow with porous and thin walls. Such structural differences led to a significant difference in porosity.

### Adhesion and proliferation on HPS and NHPS scaffolds

3D-printed PCL scaffolds are nontoxic, slowly degrading and biocompatible, so they are widely used in bone tissue engineering [34, 35]. The CCK-8 results for BMSCs (Fig. 5a) showed that cell proliferation of HPS scaffold was significantly greater than that of NHPS scaffolds on each day of measurement. We speculate that the perfusable and permeable features of HPS scaffolds, as well as their higher porosity, contribute to the higher cell proliferation levels. Previous studies have demonstrated that improved porosity has a positive impact on cell adhesion and proliferation, and thus *in vivo* bone regeneration [36, 37]. In addition, HPS scaffolds had channels that are open and interconnected from different directions, which may also be beneficial for cell attachment and proliferation.

The SEM images of BMSCs adhered to HPS scaffolds (Fig. 3d) and NHPS scaffolds (Fig. 3k) after incubation for 7 days revealed that the cell numbers and densities were much higher for HPS scaffolds. Furthermore, the SEM images revealed the close cell–cell contact of BMSCs in HPS scaffolds. In particular, the BMSCs adhered to the inner surfaces of the hollow struts and proliferate. In contrast, few cells were observed on the surfaces of the NHPS scaffolds.

**Figure 4.**
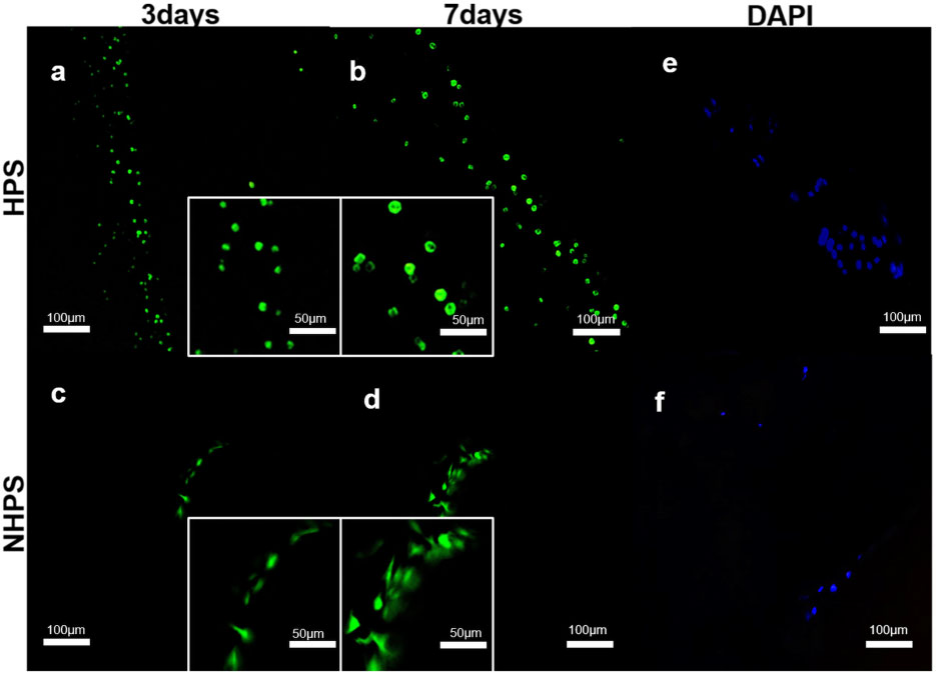
CLSM images of BMSC live/dead staining. (**a** and **b**) CLSM images of an HPS scaffold at Day 3 and Day 7, respectively. (**c** and **d**) CLSM images of an NHPS scaffold at Day 3 and Day 7, respectively. (**e** and **f**) DAPI staining images of HPS and NHPS scaffolds, respectively.

To further assess cell viability, live/dead staining was performed. As shown in Fig. 4, after culturing for 3 days, dense and numerous BMSCs remained alive along the HPS scaffold struts. Almost no dead cells were observed, and more living cells were observed on Day 7 on HPS scaffolds. By comparison, much less cells were observed on NHPS scaffolds on both Day 3 and Day 7. Consistent with live/dead staining, the result of DAPI also confirmed more cells on HPS scaffolds.

SEM and confocal laser scanning microscopy images clearly revealed the improved attachment and proliferation of BMSCs in HPS scaffolds compared to NHPS scaffolds. Thus, we can infer that HPS scaffolds provide a better microenvironment for BMSCs to adhere and proliferate. These results suggest that the integrated and interconnected hollow channels and porous channel walls in HPS scaffolds are beneficial for oxygen and nutrient distribution through the scaffolds and thus cell adhesion, spread and proliferation are improved.

### Osteogenesis and angiogenesis in HPS scaffolds

**Figure 5.**
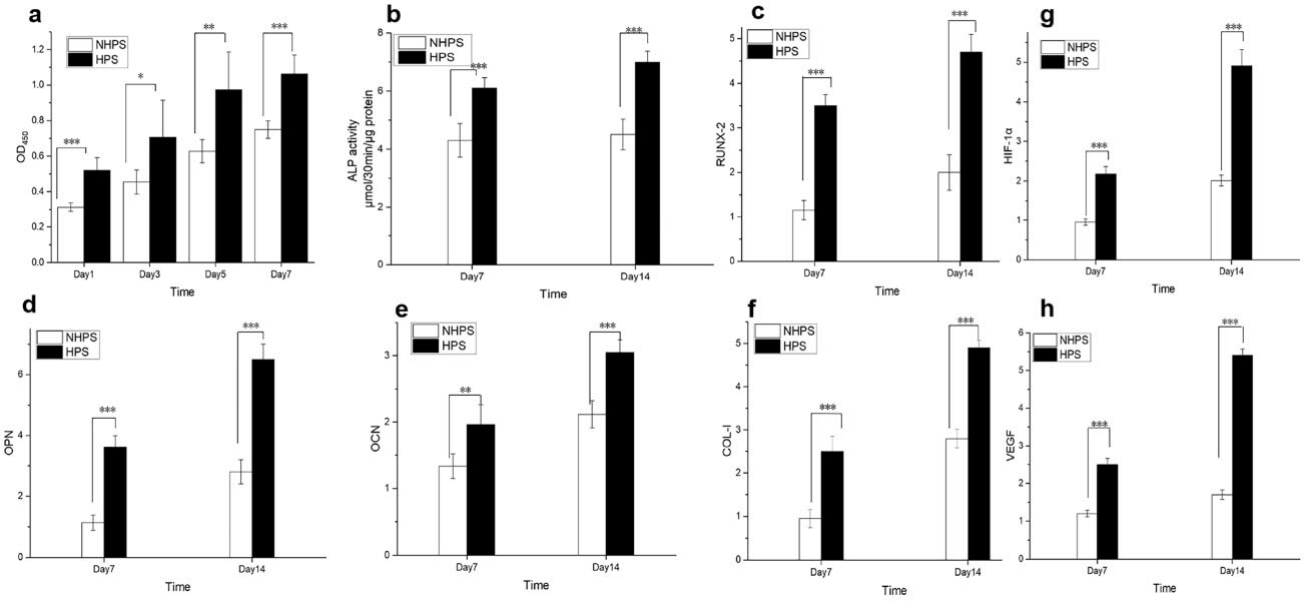
Proliferation, osteogenic differentiation and angiogenic effects of HPS and NHPS scaffolds. (**a**) CCK-8 assay results for BMSCs cultured on scaffolds at different time points. (**b**) ALP activity of BMSCs on Day 7 and Day 14. (**c**–**f**) Expression of osteogenic differentiation-related genes after incubation for 7 days and 14 days, (c) *RUNX2*, (d) *OPN*, (e) *OCN* and (f) *COL-I*. (**g** and **h**) Angiogenic effects of HPS and NHPS scaffolds on HUVECs, (g) *HIF-1α*, (h) VEGF (*n* = 6 for each group, *P < 0.05, **P < 0.01, ***P < 0.001).

**Figure 6.**
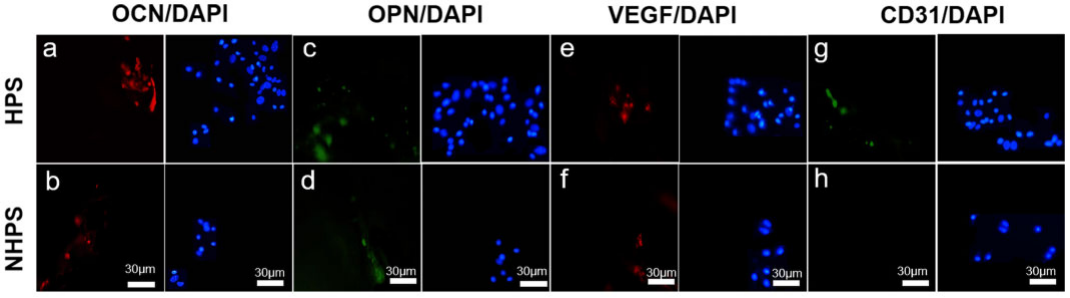
Immunofluorescence staining images of osteogenesis and angiogenesis. (**a** and **b**) OCN, (**c** and **d**) OPN, (**e** and **f**) VEGF, (**g** and **h**) CD31 after incubation for 7 days on HPS and NHPS scaffolds.

As shown in Fig. 5, the expression of osteogenesis-related genes was significantly upregulated on HPS scaffolds on both Day 7 and Day 14 compared to that on NHPS scaffolds. Moreover, HPS scaffolds significantly improved ALP activity, which indicated early mineralization and osteogenic differentiation in the BMSCs. Immunofluorescence staining of OCN and OPN further confirmed osteogenesis was remarkably enhanced on HPS scaffolds (Fig. 6).

The upregulation of bone-related genes may be due to the fact that the dense cell areas lead to increased opportunities for cell–cell contact, which facilitates osteoblastic differentiation [38]. The SEM images of BMSCs adhered to HPS scaffolds clearly revealed higher cell density and number as well as close cell–cell contact between the BMSCs. Next, we investigated the angiogenic effect of HPS scaffolds on HUVECs. HIF1-α is essential for both angiogenesis and osteogenesis, and it activates VEGF, an important angiogenic factor that can attract blood vessels [39–41]. The RT-PCR results in Fig. 5 demonstrated that *HIF1-α* gene expression of HPS scaffolds was significantly higher than that of NHPS scaffolds on both Day 7 and Day 14. Similarly, *VEGF* expression was also remarkably upregulated on HPS scaffolds on both Day 7 and Day 14. In addition, Immunofluorescence staining of VEGF and CD31 further confirmed angiogenesis was obviously enhanced on HPS scaffolds compared to NHPS scaffolds (Fig. 6). It has been reported that endothelial cells are more likely to interact with surfaces that have high porosity [42, 43]. Moreover, previous studies demonstrated that interconnected pores enhance the formation of vascular networks and offer channels for the distribution of ions, nutrients and cells [25]. Thus, the improved cell adhesion and proliferation and unique structure of HPS scaffolds could contribute to angiogenesis. In summary, the 3D-printed HPS scaffolds facilitate osteogenesis and angiogenesis *in vitro*.

### *In vivo* evaluation

**Figure 7.**
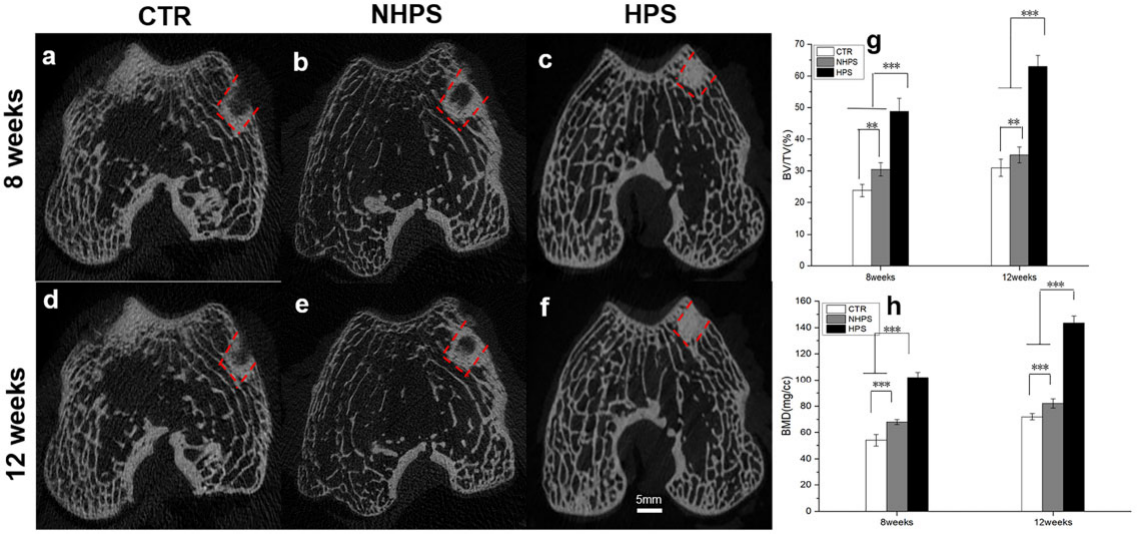
Micro-CT analysis of bone regeneration in defects at 8 and 12 weeks. (**a** and **d**) Micro-CT images for the CTR group. (**b** and **e**) Micro-CT images for the NHPS group. (**c** and **f**) Micro-CT images for the HPS group. (**g**) Micro-CT analysis of bone volume fraction (BV/TV). (**h**) Micro-CT analysis of bone mineral density (BMD).

The Micro-CT images in Fig. 7 showed that the defects in the CTR group remained mostly empty at 8 weeks and were not fully regenerated at 12 weeks, with only limited new bone formation at the edge surrounding the defect when observed in the coronal view. For the NHPS group, new bone was formed at the edge surrounding the scaffold and limited new bone formation in the inner part of the porous scaffold was observed in the coronal view at 12 weeks. However, for the HPS group, new bone ingrowth was observed throughout the whole defect. At 8 weeks, extensive bone formation was observed in the inner part of the scaffold, and more bone regeneration was observed at 12 weeks. The Micro-CT images indicated that HPS scaffolds not only stimulated new bone tissue formation around the scaffold but also facilitated bone formation in the core of the scaffolds. In addition, the BV/TV and BMD values for the HPS group were significantly higher than those for the NHPS and CTR groups at both time points. Therefore, the unique structure of the HPS scaffolds promotes bone regeneration more effectively.

**Figure 8.**
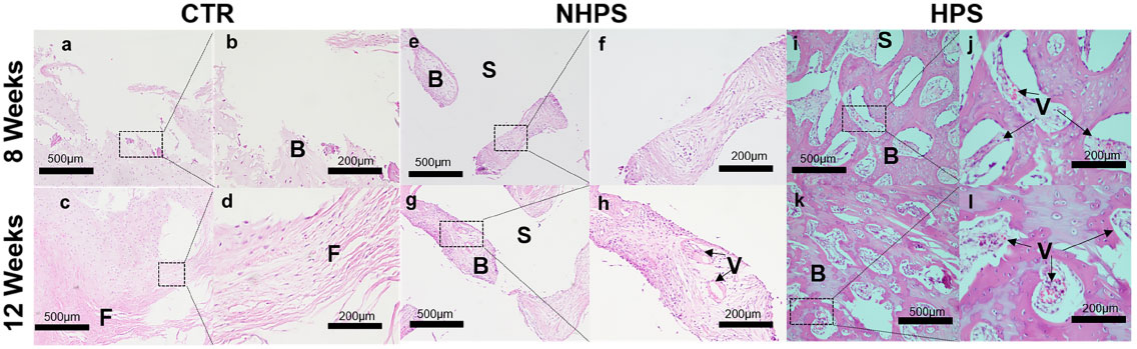
HE staining showing bone regeneration *in vivo* at 8 and 12 weeks after implantation. (**a**–**d**) CTR (control group), (**e**–**h**) NHPS group and (**i**–**l**) HPS group. (F: fibrous tissues, B: new bone tissue, S: scaffold, V: new vessels).

**Figure 9.**
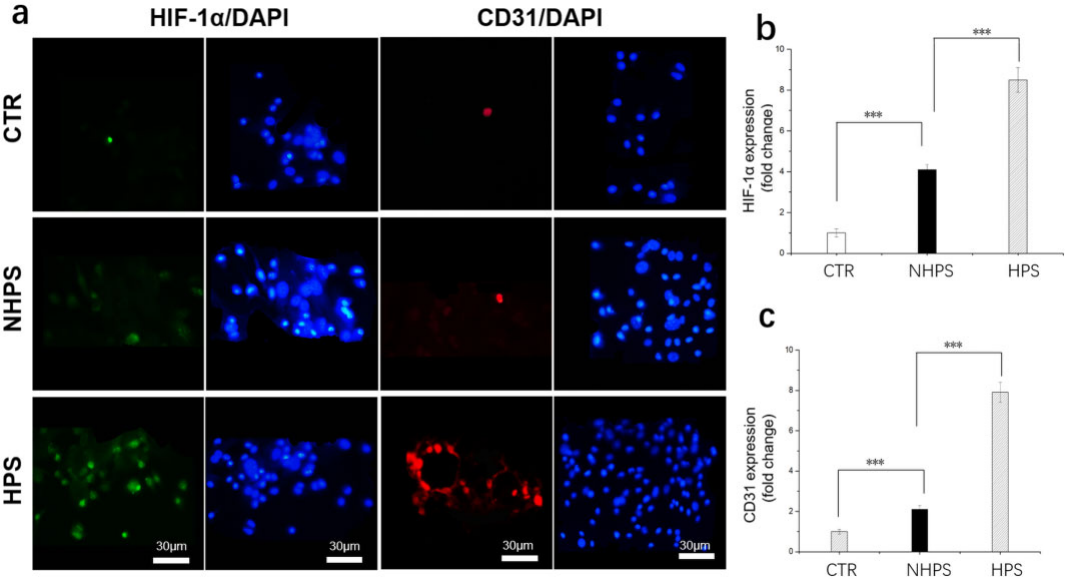
(**a**) Representative immunofluorescence staining of *in vivo* samples at 12 weeks, (**b** and **c**) summarized data showing the difference of protein expression in scaffolds group compared to control group (*n* = 6 for each group, ***P < 0.001).

The HE staining images in Fig. 8 revealed that new bone formation for HPS group was significantly more extensive than that in the NHPS and CTR group. Importantly, there were more newly formed vessels and osteoblasts in the HPS scaffold pores compared to NHPS and CTR group. Red blood cells, which facilitate bone formation, could be observed in the newly formed vessels in the HPS scaffold. In addition, some parts of the PCL struts in the HPS scaffold had degraded, while the slow degradation of NHPS scaffold restrained new bone growth in the defect. Comparatively, fibrous tissues were observed in the CTR defects. Moreover, we also observed higher expression of both HIF-1α and CD31 in the HPS scaffold than NHPS scaffold based on immunofluorescence staining images (Fig. 9). HPS scaffolds are vascular-like scaffolds, which contain plenty of interconnected channels. Vascular-like scaffolds have been demonstrated to be a successful strategy to enhance rapid vascularization both *in vitro* and *in vivo*, which further improve formation of new bone tissue [18, 23, 44]. Channels that resemble physiologic microvasculature support vascularization. In addition, compared with NHPS scaffolds, HPS scaffolds have a higher porosity and rougher texture, which contribute to osteogenesis [45, 46]. In summary, the highly interconnected structure and permeable walls of the HPS scaffolds promote vascularization and bone regeneration *in vivo*.

## Conclusions

In this study, we fabricated perfusable and permeable PCL scaffolds with highly interconnected hollow channels via an indirect one-pot 3D-printing method. The unique interconnected HPS scaffolds significantly enhance cell adhesion, spread and proliferation as well as osteogenic differentiation and angiogenesis. Thus, these structures show promise for application in cell delivery and bone regeneration. In addition, this strategy can be used to prepare other polymer scaffolds, indicating its potential for tissue engineering, mechanical engineering, catalysis and environmental remediation.

## Funding

This work was supported by the National Natural Science Foundation of China (82072400, 82102211, 52173117), the Natural Science Foundation of Jiangsu Province (BK20200001), the Natural Science Foundation of Shanghai (20ZR1402500), the Belt & Road Young Scientist Exchanges Project of Science and Technology Commission Foundation of Shanghai (20520741000), Ningbo 2025 Science and Technology Major Project (2019B10068), the Science and Technology Commission of Shanghai Municipality (20DZ2254900, 20DZ2270800), the Fundamental Research Funds for the Central Universities, DHU Distinguished Young Professor Program (LZA2019001).

*Conflicts of interest statement*. None declared.
